# Stress fluctuations and adiabatic speed of sound in liquids: a simple way to estimate it from ab initio simulations

**DOI:** 10.1038/s41598-023-45338-2

**Published:** 2023-10-23

**Authors:** Taras Bryk, Giancarlo Ruocco, Ari Paavo Seitsonen

**Affiliations:** 1 Institute for Condensed Matter Physics of National Academy of Sciences of Ukraine, Lviv, 79011 Ukraine; 2 Institute of Applied Mathematics and Fundamental Sciences, Lviv National Polytechnic University, Lviv, 79013 Ukraine; 3 Center for Life Nano Science @Sapienza, Istituto Italiano di Tecnologia, 00161 Rome, Italy; 4 Dipartimento di Fisica, Universita’ di Roma “La Sapienza”, 00185 Rome, Italy; 5 Département de Chimie, École Normale Supérieure, 75005 Paris, France

**Keywords:** Chemical physics, Condensed-matter physics

## Abstract

One of the fundamental quantities in dynamics of the liquid state, the adiabatic speed of sound $c_s$, is extremely difficult to predict from computer simulations, especially in ab initio simulations. Here we derive an expression for the instantaneous correlator of fluctuations of longitudinal component of stress tensor, which contains $c_s$ along with others quantities easy accessible via classical and ab initio computer simulations. We show that the proposed methodology works well in the case of Lennard-Jones and soft-sphere simple fluids, Kr–Ar liquid mixture in connection with simulations with effective pair interactions as well as for liquid Sb, fluid Hg and molten NaCl from ab initio simulations.

## Introduction

Sound propagation and its different mechanisms in liquids and solids are of paramount importance for fundamental many-particle effects in condensed matter, as well as for technologies based on vibrational properties of crystals and disordered systems. The difference of acoustic long-wavelength propagating modes in liquids from standard phonons in crystals is well known^1,2^. In the low, $\omega \rightarrow 0$ limit, at variance with the crystals, where the displacements around stable potential energy minima are described via propagating longitudinal (L) and transverse (T) waves, in the case of liquids one has the absence of long-wavelength transverse sound and a pressure wave with adiabatic propagation speed in longitudinal case^3,4^. The adiabatic sound propagation is a very specific feature of the liquid state, when on macroscopic scales the pressurized and rarefied regions of the pressure wave have different local temperatures as the thermal diffusion is always slower than the wave oscillation period, ($D_Tk^2\ll c_sk$), and the local thermal equilibrium cannot be reached. Here $D_T$ is the thermal diffusivity, *k* - the wave number and $c_s$ the adiabatic speed of sound. Only at much higher *k*, when $D_Tk^2\approx c_sk$, the local equilibrium is guaranteed^5^. On macroscopic scale, however, the fluctuations of local temperature cause an increase of the propagation speed in comparison with the isothermal case ($c_T$) by $\gamma ^{1/2}$ times with $\gamma$ being the ratio of specific heats. By definition the adiabatic speed of sound is defined by the isothermal compressibility and ratio of specific heats^6,7^.

1 $$\begin{aligned} c_s= \left[ \frac{B_s}{\rho } \right] ^{1/2}= \left[ \left( \frac{\partial P}{\partial \rho } \right) _s \right] ^{1/2}= \left[ \gamma \left( \frac{\partial P}{\partial \rho } \right) _T \right] ^{1/2}\equiv \sqrt{\gamma }c_T~. \end{aligned}$$

here $B_s$ is the adiabatic bulk modulus, *P*—pressure and $\rho$—mass density.

It is a highly non-trivial task to predict the macroscopic adiabatic speed of sound in fluids from computer simulations. Expressing $c_s$ via quantities calculated in molecular dynamics (MD) simulations one has

2 $$\begin{aligned} c_s= \left[ \frac{\gamma k_BT}{mS(0)} \right] ^{1/2}=\lim _{k\rightarrow 0} \left[ \frac{\gamma (k)}{S(k)} \right] ^{1/2}v_{th}, \end{aligned}$$

where $S(k)=\langle n_{-k}n_{k}\rangle$ is the structure factor (static density-density correlator), $k_B$—Boltzmann constant, *T*—temperature and $v_{th}$—the average thermal speed. The *k*-dependent ratio of specific heats $\gamma (k)$ can be estimated via computer simulations from static correlators $\langle e_{-k}e_{k}\rangle$, $\langle n_{-k}e_{k}\rangle$ and $\langle {\dot{J}}^L_{-k}e_{k}\rangle$^8–10^.

It is practically impossible to estimate correctly the $c_s$ from the calculated dispersion law of acoustic branch $\omega (k)$, because in simulations the smallest sampled wave number compatible with periodic boundary conditions is $k_{min}=2\pi /L$ with *L* being the box length, and there is no guarantee that $k_{min}$ reached the hydrodynamic region^3^. At the boundary of hydrodynamic regime^11^ the dispersion law changes from macroscopic linear dependence^12,13^ and depending on the coupling between density, heat and stress fluctuations can show either positive (positive sound dispersion) or negative (negative sound dispersion) deviation from macroscopic hydrodynamic law^14^. Therefore the primitive linear extrapolation of the dispersion law $\omega (k)$ towards $k=0$ will not result in correct value of $c_s$.

There exist three basic approaches to calculation of $c_s$ from computer simulations. The first one is based on estimation of the equation of state *F*(*P*, *V*, *T*)^15^, fitting it to some analytical form, and estimation of the isothermal compressibility. Weak points here are in many runs for estimation of *F*(*P*, *V*, *T*) at fixed temperature or pressure and in the need to have additionally the value of $\gamma$, which is taken either from experiments, or from thermodynamic relation for the difference of specific heats $C_P-C_V$ via derivatives $(\partial P/\partial T)_V$ and $(\partial P/\partial V)_T$, that requires very precise knowledge of the equation of state in the ranges of interest for P,V,T. Another possibility is to perform separate simulations in (NVT) and (NPT) ensembles in order to estimate separately $C_V$ and $C_P$ and consequently $\gamma$^16^.

The second approach was proposed by Lustig^17^ and consists in estimation of a number of ensemble averages $\langle U\rangle$, $\langle U^2\rangle$, $\langle \frac{\partial U}{\partial V}\rangle$, $\langle (\frac{\partial U}{\partial V})^2\rangle$, $\langle \frac{\partial ^2 U}{\partial V^2}\rangle$, which in dfferent combinations result in macroscopic values for isobaric heat capacity $C_p$, adiabatic speed of sound $c_s$, and Joule-Thomson coefficient $\mu _{JT}$^17^. Here *U* is the potential energy of the system and *V* - its volume. This methodology was implemented in the software package *ms2*^18^ for molecular simulations with effective interatomic potentials. So far it is unclear whether this methodology could be applied with ab initio simulations, when the subsystem of valence electrons is treated explicitly via density functional theory and total energy of the system is a complex functional of electron density^19^.

The third approach is easily applicable with classical (with interatomic effective interactions) MD simulations and needs calculations of *k*-dependent correlators involving energy (or heat) density^10,20^, that allows estimation of *k*-dependent ratio of specific heats $\gamma (k)$. The ratio $\gamma (k)/S(k)$ as a function of wave numbers is a smooth function that allows reasonable extrapolation towards $k\rightarrow 0$ to use in Eq. (2). The precision of this approach is much better than for the first one and it allows to determine $c_s$ with an uncertainty of order 7–8%^21,22^. However, in the case of ab initio (with explicit treatment of electron subsystem via density functional theory) simulations the second approach is difficult to apply, because of huge efforts to sample *k*-dependent energy fluctuations within the DFT. In^23^ it was proposed to avoid sampling of *k*-dependent energy fluctuations by using a simultaneous fit of theoretical (within the thermo-viscoelastic (TVE) model) density-density and current-current time correlation functions to the corresponding AIMD-derived ones. The fitting parameters being correlators involving energy fluctuations allowed the calculation of $\gamma (k)$ and corresponding ratio $\gamma (k)/S(k)$, however such a fitting results in much lower precision in estimation of the adiabatic speed of sound, than directly from classical simulations. Hence, any new approach to estimate the adiabatic speed of sound from classical and *ab intio* simulations avoiding complicated calculations of $\gamma (k)$ will be of great interest. In this paper we present an alternative approach that allows to determine $c_s$ starting from an easy-to-calculate stress tensor corellation function and other accessible quantities.

The remaining part of the paper is organized as follows. In the next Section we report an expression, which allows calculations of the adiabatic speed of sound via the instantaneous averages of the macroscopic longitudinal stress and high-frequency speed of sound. The proposed methodology is applied then to calculations of $c_s$ for Lennard-Jones, soft sphere (purely repulsive) fluids and a binary liquid mixture, as well as for fluid Hg, liquid Sb and NaCl from ab initio simulations.

## Results

### Theory

The generalized hydrodynamics in long-wavelength limit must be consistent with the Green-Kubo expressions for macroscopic transport coefficients because they describe the same relaxation phenomena. So, the *k*-dependent shear-stress relaxation time in generalized hydrodynamic description of transverse collective dynamics in liquids

3 $$\begin{aligned} \tau ^T_{stress}(k)= \left[ \frac{G_{\infty }}{\eta }-\frac{\eta }{\rho }k^2 \right] ^{-1} \end{aligned}$$

tends in the macroscopic limit $k\rightarrow 0$ to the Maxwell relaxation time^24^.

$$\begin{aligned} \tau ^T_{stress}(k\rightarrow 0)\equiv \tau _M=\frac{\eta }{G_{\infty }}. \end{aligned}$$

In Eq. (3) $\eta$, $G_{\infty }$ and $\rho$ are the shear viscosity, high-frequency shear modulus and mass density, respectively. It is important to emphasize that the Maxwell relaxation time is in fact the correlation time of macroscopic shear-stress autocorrelation functions with the standard Green-Kubo integral

$$\begin{aligned} \tau _M=\frac{1}{\psi (0)}\int _{0}^{\infty }\psi (t) dt\equiv \tau _{corr}~, \end{aligned}$$

where

$$\begin{aligned} \psi (t)=\frac{V}{k_BT}\langle \sigma _{xy}(t)\sigma _{xy}(0)\rangle ~, \end{aligned}$$

$\sigma _{\alpha \beta }$ are the Cartesian components of stress tensor, and

$$\begin{aligned} G_{\infty }\equiv \frac{V}{k_BT}\langle \sigma _{xy}(0)\sigma _{xy}(0)\rangle ~. \end{aligned}$$

For longitudinal collective dynamics the relaxation of diagonal stress tensor components is different^14,25,26^ than the relaxation of off-diagonal components (for the transverse case) due to interaction of longitudinal stress fluctuations with density and heat ones:

4 $$\begin{aligned} \tau ^L_{stress}(k)= \left[ \frac{c^2_{\infty }-c^2_s}{D_L}- \left( D_L- (\gamma -1)\Delta \right) k^2 \right] ^{-1}, \end{aligned}$$

where $D_L=(\eta _{b}+4\eta /3)/\rho$ is the longitudinal kinematic viscosity with $\eta _b$ being bulk viscosity, $c_{\infty }=[(B_{\infty }+4G_{\infty }/3)/\rho ]^{1/2}$ is the high-frequency speed of “bare” (non-damped) acoustic-like excitations in liquid, and $B_{\infty }$ is the high-frequency bulk modulus. The term with a prefactor $(\gamma -1)$ in the right hand side of (4) is due to coupling of stress tensor with heat fluctuations, which is characterized by a correlator $\Delta$^14^. Since we are interested in the macroscopic limit of (4), the explicit expression of the latter quantity is of no importance here, and

5 $$\begin{aligned} \tau ^L_{stress}(k\rightarrow 0)=\frac{D_L}{c^2_{\infty }-c^2_s}~, \end{aligned}$$

and as in the transverse case it should be the correlation time of the macroscopic autocorrelation function

6 $$\begin{aligned} \tau ^L_{corr}=\frac{1}{\psi ^L(0)}\int _{0}^{\infty }\psi ^L(t) dt~, \end{aligned}$$

where

$$\begin{aligned} \psi ^L(t)=\frac{V}{k_BT}\langle (\sigma _{zz}(t)-\bar{P}) (\sigma _{zz}(0)-\bar{P})\rangle ~, \end{aligned}$$

$\bar{P}$ is the average pressure over the production run. Equalizing (5) and (6), and making use of the Kubo relation for longitudinal stress autocorrelation function^1^ we obtain a relation

7 $$\begin{aligned} \psi ^L(0) \equiv B_{\infty }+4G_{\infty }/3-B_s=\rho (c_{\infty }^2-c_s^2)~, \end{aligned}$$

which connects the instantaneous correlator of longitudinal stress tensor fluctuations with the difference of squares of high-frequency and adiabatic speeds of sound, and $B_s$ being the adiabatic bulk modulus. The nice feature of this expression is the possibility to estimate the adiabatic speed of sound from the values of the high-frequency speed of sound and instantaneous correlator of longitudinal stress fluctuations:

8 $$\begin{aligned} c_s=\sqrt{c_{\infty }^2-\psi ^L(0)/\rho }~, \end{aligned}$$

i.e. without explicit need of $\gamma$ or sampling the energy (or heat) fluctuations from MD. The high-frequency speed of sound can easily be calculated from classical^27^ or ab initio^23,28^ simulations as

9 $$\begin{aligned} \lim _{k\rightarrow 0} \frac{\left\langle {\dot{J}}^L(-k){\dot{J}}^L(k) \right\rangle }{\left\langle J^L(-k)J^L(k) \right\rangle } = c_{\infty }^2k^2~, \end{aligned}$$

where ${\dot{J}}^L(k)$ is the first time derivative of spatial Fourier-components of the longitudinal part $J^L(k)$ of mass-current $\textbf{J}(k)$, defined as

10 $$\begin{aligned} \textbf{J}(k,t)=\frac{1}{\sqrt{N}}\sum _{j=1}^{N}m_j\textbf{v}_j(t)e^{-i\textbf{kr}_j(t)}~, \end{aligned}$$

and its first time derivative being

11 $$\begin{aligned} \frac{d\textbf{J}(k,t)}{dt}\equiv {\dot{\textbf{J}}}(k,t)=\frac{1}{\sqrt{N}}\sum _{j=1}^{N} [\textbf{F}_j(t)-im_j(\textbf{kv}_j)\textbf{v}_j(t)]e^{-i\textbf{kr}_j(t)}~, \end{aligned}$$

where $m_j$ is the atomic mass of the *j*-th particle, $\textbf{r}_j(t)$, $\textbf{v}_j(t)$ and $\textbf{F}_j(t)$ are the trajectory, velocity and force acting on the *j*-th particle. Hence, using $c_{\infty }$ from (9) we can calculate $c_s$ from (8). One can easily sample (10) and (11) in classical or ab initio simulations. Having (10) and (11) one has to calculate the averages in (9) and obtain $c_{\infty }$ from (9). Then one needs the values of components of stress tensor along the production run and evaluate from them the $\psi ^L(0)$. In the end, one uses the relation (8) to obtain $c_s$ from $\psi ^L(0)$ and $c_{\infty }$.

### Application of the proposed methodology

We will check the adiabatic speed of sound calculated via expression (8) for supercritical Ar at T = 280 K and a soft sphere fluid in connections with classical MD simulations and comparison with previously calculated values of $c_s$ via heat fluctuations (2). An application to liquid mixtures via classical simulations is performed on liquid equimolar mixture KrAr at temperature 116 K. In order to show that expression (8) can be used with ab initio simulations we apply it to the calculation of the high-frequency and adiabatic speeds of sound and compare for liquid Sb at 793 K, which recently was studied by inelastic X-ray scattering^29^, then for the case of fluid mercury at T = 1750 K and for molten NaCl at 1262 K.

**Figure 1:**
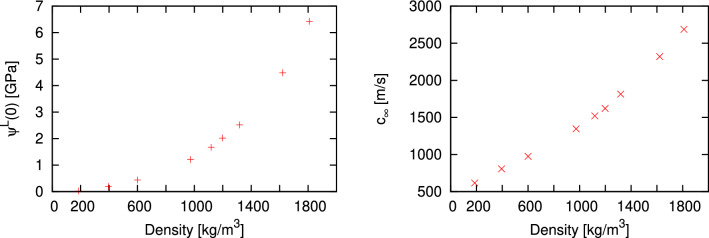
Density dependence of the instantaneous longitudinal stress autocorrelation $\psi ^L(0)$ and high-frequency speed of sound $c_{\infty }$ used in Eq. (8) for supercritical Ar at temperature 280 K.

In Fig. 1 we show the density dependence of two main quantities $\psi ^L(0)$ and $c_{\infty }$ needed for calculations of the adiabatic speed of sound. We would like to remind that the high-frequency speed of sound $c_{\infty }$ is the characteristic idealized speed of non-damped long-wavelength longitudinal waves in elastic continuum having the bulk $B_{\infty }$ and shear $G_{\infty }$ moduli. Both calculated dependences in Fig. 1 show monotonic increase with density.

**Figure 2:**
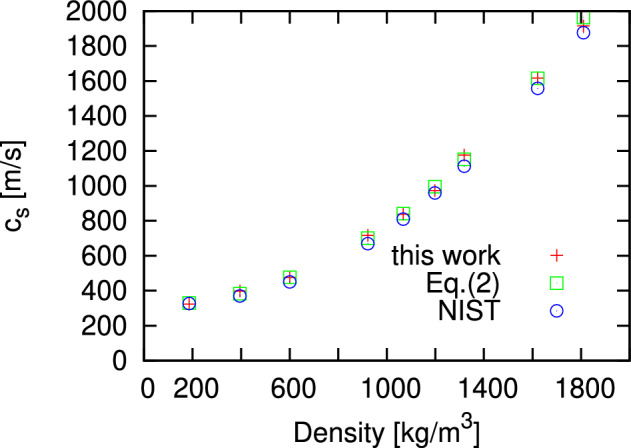
Density dependence of the adiabatic speed of sound in supercritical Ar at T = 280 K obtained from the proposed methodology (plus symbols) in comparison with the $c_s$ obtained via Eq. (2) (open boxes) and NIST experimental data^30^ (open circles).

Combining the density dependence in Fig. 1 according to (8) one obtains the adiabatic speed of sound $c_s$ as a function of density at T = 280 K as shown in Fig. 2. For comparison by open boxes we show the results of estimation of $c_s$ via the long-wavelength limit of the ratio of *k*-dependent quantities $\gamma (k)$ and *S*(*k*) according to the right hand side of Eq. 2. The latter was calculated in a sophisticated way via sampling of *k*-dependent energy- (or heat-)density fluctuations in MD simulations and calculations of corresponding static correlators. One can see that there is practically good agreement of $c_s$ obtained by the proposed methodology via Eq. (8) and via the previous sophisticated methodology via Eq. (2). Both sets of results are in good agreement with the NIST database for supercritical Ar at 280 K.

**Figure 3:**
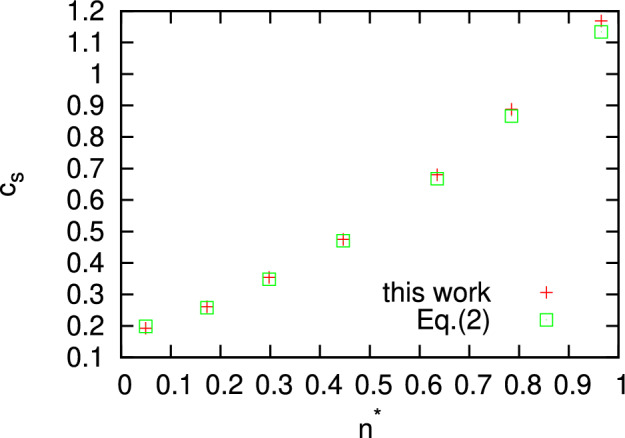
Density dependence of the adiabatic speed of sound in soft sphere fluids obtained from the proposed methodology and compared with the $c_s$ obtained via *k*-dependent ratio of specific heats and structure factor (2)^27^. The standard reduced units for soft core systems as in^27^ were used.

The other class of simple model fluids, which do not have interparticle attraction, the soft sphere fluids, were simulated for seven densities at thermodynamic conditions and with simulation setup identically as in^27^. Again, the adiabatic speed of sound via Eq. (8) is in nice agreement (see Fig. 3) with the results of^27^ obtained via Eq. (2) and sophisticated sampling of *k*-dependent energy-density fluctuations.

**Figure 4:**
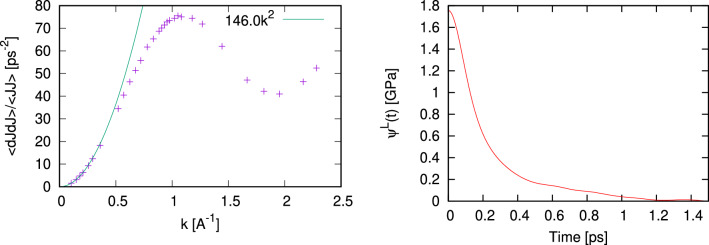
The wave number dependence of $\frac{\langle {\dot{J}}^L(-k){\dot{J}}^L(k)\rangle }{\langle J^L(-k)J^L(k)\rangle }$ from Eq. (9) for a binary KrAr liquid at density 1.832 g/cm$^3$ and temperature 116 K (**a**) and autocorrelation functions of longitudinal (L) and transverse (T) stress fluctuations (**b**).

Here we show an evidence that the proposed methodology can be easily applied to the case of many-component systems and in particular, to a binary equimolar liquid mixture KrAr at T = 116 K. The classical simulations with 4000 particles in microcanonical ensamble and production run of 360,000 time steps allowed to obtain well converged static averages for calculations of quantities entering (8). In Fig. 4 we show how perfectly well the wave-number dependence of $\frac{\langle {\dot{J}}^L(-k){\dot{J}}^L(k)\rangle }{\langle J^L(-k)J^L(k)\rangle }$ corresponds to the $k^2$ asymptote in the long-wavelength region. Note, that $J^L(k,t)$ are the space-Fourier components of longitudinal part of total mass current, which includes both components according to (10). Another quantity, $\psi ^L(0)$ was well converged and estimated from the stress autocorrelation function at $t=0$ (see Fig. 4b). Inserting these numbers obtained from MD simulations in (8) one obtains a prediction for the adiabatic speed of sound in equimolar liquid mixture KrAr at T = 116 K to be 722.03 m/s. We can compare this macroscopic value with the eigenfrequencies of the generalized Langevin equation for liquid KrAr at the same thermodynamic point and simulated with rather small number of particles 864^31^. For the smallest *k*-value in that study, which was outside the hydrodynamic region, the phase speed of sound excitations obtained from the eigenfrequency was 808 m/s^31^, that is a consequence of positive sound dispersion outside the hydrodynamic regime.

## Discussion

We have shown above that the proposed methodology works perfectly with classical molecular dynamics simulations. The great advantage of the proposed methodology that in contrast to the huge efforts in estimation of $c_s$ via Eq. 2 it can be applied to ab initio simulations. However, a weak point of the ab initio simulations is in rather small size of the simulated system. We will show below how the proposed methodology works in the case of simulated systems with 600 (liquid Sb), 200 (fluid Hg) and 300 (molten NaCl) particles.

**Figure 5:**
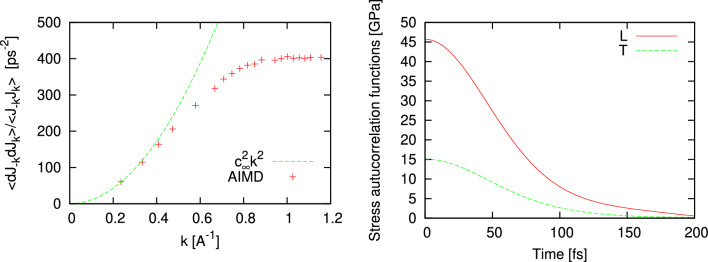
The wave number dependence of $\frac{\langle {\dot{J}}^L(-k){\dot{J}}^L(k)\rangle }{\langle J^L(-k)J^L(k)\rangle }$ from Eq. (9) for molten Sb at density 6.43 g/cm$^3$ and temperature 973 K (**a**) and autocorrelation function of longitudinal stress fluctuations (**b**).

The ab initio simulations for liquid Sb at 973 K were performed with VASP package using 600 particles over the production run of 17,700 configurations. Projector-augmented-wave (PAW) potentials^32,33^ with 3 valence electrons were used for electron-ion interaction. The exchange-correlation functional was taken in the Perdew-Burke-Ernzerhof (PBE) GGA formulation^34^ and the Brillouin zone sampling was restricted by the single $\Gamma$ point. In Fig. 5 we show the *k*-dependence of $\frac{\langle {\dot{J}}^L(-k){\dot{J}}^L(k)\rangle }{\langle J^L(-k)J^L(k)\rangle }$ and expected $k^2$-dependence crossing the point at the smallest available from AIMD *k*-value. The value $\psi ^L(0)$ from the calculated stress autocorrelations is equal to 45.718 GPa, and making use of Eq. (8) results in the estimated value $c_s$ = 1923.9 m/s. The experimental data for the adiabatic speed of sound was taken from^29^ as 1910 m/s and from^35^ where four sets of experimental data for liquid Sb were fitted to a polynomial $c(T)=1330.6+1.0471 T - 4.49466\cdot 10^{-4}T^2$ giving 1933.1 m/s at 973 K. Hence, a good agreement of our estimated value of $c_s$ with the experimental data is observed.

Much sophisticated for ab initio simulations is the case of fluid mercury because of metal-nonmetal transition which takes place at density $\sim$ 9 g/cm$^3$. The effective electron-ion interaction should explicitly treat ten 5d electrons, that results in very large number of electron states to be converged at each step of AIMD and usually the reported simulation studies were performed with rather small number of atoms: 50 mercury atoms in^36^, 90 atoms in^37,38^, while recently there appeared an AIMD study with a comparison of results performed with 100, 200 and 400 mercury atoms^39^. We performed AIMD with 200 Hg atoms and PAW potentials with explicitly treated 12 (ten 5d and two 6s-p) electrons. The density of our system was 10.0 g/cm$^3$ and temperature was kept at 1750 K. We performed two sets of simulations: one with default plane-wave cut-off energy for VASP library PAW potentials (233.2 eV) and another one with the cut-off energy of 340 eV. The production runs were 20,000 timesteps for each of these two different plane-wave energy cut-offs, respectively.

**Figure 6:**
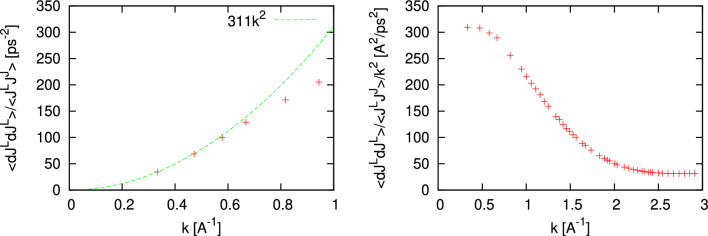
The wave number dependence of $\frac{\langle {\dot{J}}^L(-k){\dot{J}}^L(k)\rangle }{\langle J^L(-k)J^L(k)\rangle }$ from Eq. (9) for liquid Hg at density 10.0 g/cm$^3$ and temperature 1750 K (**a**) and its long-wavelength asymptote divided by $k^2$ (**b**).

In Fig. 6 we show that 200 particles (default VASP cut-off for plane-wave expansion) for liquid Hg are sufficient to recover almost perfect $k^2$ long-wavelength dependence of $\frac{\langle {\dot{J}}^L(-k){\dot{J}}^L(k)\rangle }{\langle J^L(-k)J^L(k)\rangle }$, which results in the high-frequency speed of sound $c_{\infty }$ = 1763.5 m/s. Corresponding value of $\psi ^L(0)$ for this simulation was 17.474 GPa, that results via (8) in the adiabatic speed of sound $c_s$ = 1167.3 m/s. Increasing the plane-wave cut-off energy up to 340 eV results in the following numbers: $c_{\infty }$ = 1784.7 m/s, $\psi ^L(0)$ = 17.629 GPa and adiabatic speed $c_s$ = 1192.5 m/s, i.e. showing an increase of order just 2.2 percent due to much better convergence of stress tensor components with increase of plane-wave cut-off energy in wave function expansion.

**Figure 7:**
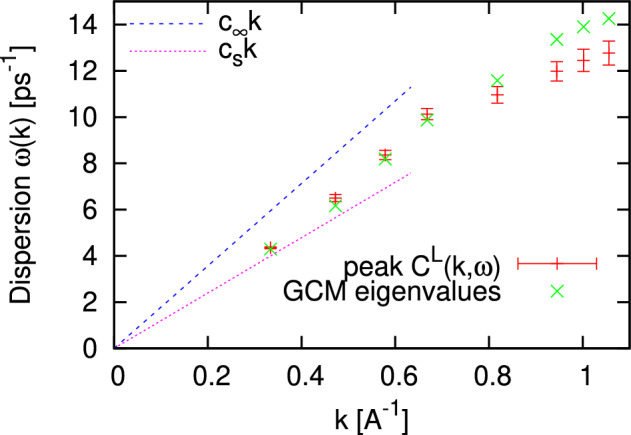
Dispersion of the collective excitations in the first pseudo-Brillouin zone for liquid Hg at density 10.0 g/cm$^3$ and temperature 1750 K. Results of numerical estimation of dispersion via the peak positions of longitudinal current spectral function $C^L(k,\omega )$—“plus” symbols with error bars, the GCM eigenvalues within the thermo-viscoelastic theory^23^—“cross” symbols. Linear dispersion laws with the adiabatic $c_s$ and high-frequency $c_{\infty }$ speeds of sound are shown by long- and short-dash lines, respectively.

In Fig. 7 we show how the high-frequency and adiabatic speeds of sound correspond to the observed dispersion of collective excitations in fluids Hg at T = 1750 K and density 10.0 g/cm$^3$. By plus symbols with error bars we show purely numerical estimation of dispersion $\omega (k)$ via peak positions of longitudinal current spectral function $C^L(k,\omega )$, calculated as Fourier transformed longitudinal mass-current autocorrelation function $F^L_{JJ}(k,t)$. In order to verify this numerical dispersion we calculated dynamic eigenmodes via the Generalized Collective Mode (GCM) approach^9,10,23^, which allows decomposition of time correlation functions into contributions from propagating (complex-conjugated pairs of eigenvalues of generalized hydrodynamic matrix) and relaxing (purely real eigenvalues) modes. The standard deviation of the peak positions of $C^L(k,\omega )$ from propagating eigenmodes for large wave numbers is the consequence of inability in the numerical procedure to separate contributions from relaxing modes which dominate in the collective dynamics with increasing wave numbers as is known for dynamic structure factors

$$\begin{aligned} S(k,\omega )=\frac{k^2}{\omega ^2}C^L(k,\omega )~. \end{aligned}$$

We see in Fig. 7 that there exists a strong deviation of the observed $\omega (k)$ from the linear hydrodynamic dispersion law, which is known as the positive sound dispersion^1,14,25^. For the case of fluid Hg this is in qualitative agreement with experimental findings for the emergence of strong positive sound dispersion in the region of metal-nonmetal transition in liquid Hg^40^.

**Figure 8:**
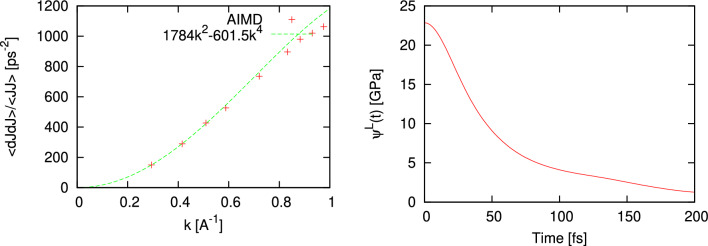
The wave number dependence of $\frac{\langle {\dot{J}}^L(-k){\dot{J}}^L(k)\rangle }{\langle J^L(-k)J^L(k)\rangle }$ from Eq. (9) for molten NaCl at density 1.4947 g/cm$^3$ and temperature 1262 K (**a**) and autocorrelation function of longitudinal stress fluctuations (**b**).

We applied the proposed methodology on adiabatic speed of sound to another binary liquid, molten NaCl, in connection with ab initio simulations. We simulated molten NaCl at density 1.4947 g/cm$^3$ and temperature 1262 K with 300 particles with similar PAW potentials for electron-ion interaction and PBE exchange-correlation. Due to rather high density the dependence $\frac{\langle {\dot{J}}^L(-k){\dot{J}}^L(k)\rangle }{\langle J^L(-k)J^L(k)\rangle }$ does not correspond well to the simple asymptote $\sim k^2$ in the long-wavelength region, therefore for the high-density systems it is possible to fit the *k*-dependence in Fig. 8 with a polynom $ak^2+bk^4$. In the limit $k\rightarrow 0$ we retain just the first term using the coefficient $a=c^2_{\infty }$. This allowed to estimate $c_{\infty }$ = 4223.74 m/s, while using $\psi ^L(0)$ = 22.865 GPa (see Fig. 8) one obtains from (8) the value $c_s$ = 1594.56 m/s, which is in reasonable agreement with the experimental values 1586.3 m/s(at T 1253 K) and 1558.9 m/s (at T = 1283K)^41^. Note, that the recent AIMD calculations of the dispersion of collective excitations in molten NaCl^42^ give evidence of a large positive dispersion in this molten salt in comparison with the macroscopic hydrodynamic dispersion law with adiabatic speed of sound estimated in the actual study.

In summary, we suggested, that the correlation time of macroscopic longitudinal stress autocorrelation function is equivalent to the long-wavelength limit of the longitudinal stress relaxation time as obtained from generalized hydrodynamics, exactly as this is in the transverse case. We arrived at Eq. (8), which connects $\psi ^L(0)$ with the high-frequency $c_{\infty }$ and adiabatic $c_s$ speeds of sound. This allows estimation of the adiabatic speed of sound from classical and ab initio simulations without the need to know the ratio of specific heats $\gamma$ or to sample *k*-dependent energy (or heat) fluctuations in simulations.

We applied the proposed methodology to calculations of the adiabatic speed of sound from classical molecular dynamics in supercritical Ar (treated as a Lennard-Jones fluid) and a soft sphere fluid. For both types of fluids we obtained perfect agreement of the proposed methodology with the previously obtained values^14,27^ of $c_s$ in a wide range of densities via Eq. (2) and sampling of *k*-dependent energy (heat) fluctuations. Also, for supercritical Ar the calculated values of adiabatic speed of sound are in good agreement with the experimental data^30^. The extension of the methodology on many-component liquids is straightforward, as it was demonstrated for the case of a binary liquid mixture KrAr.

An important point of this study is in demonstration that the proposed methodology is quite easily applicable with ab initio simulations. For liquid Sb simulated with 600 particles we obtained a perfect agreement with experimental values of the adiabatic speed of sound^35^. Also, the proposed methodology was applied to the estimation of the adiabatic speed of sound in fluid Hg at temperature 1750 K and density 10.0 g/cm$^3$, which is in metallic state. The obtained adiabatic speed of sound was used to compare the calculated from AIMD dispersion curve $\omega (k)$ of collective excitations with linear hydrodynamic dispersion law. This allowed to estimate the positive sound dispersion to be of order 25% that is in agreement with experimental findings for the emergence of strong positive sound dispersion in the region of metal-nonmetal transition in liquid Hg^40^, which takes place at the density $\sim$ 9.0 g/cm$^3$^40^. We tried to obtain the adiabatic speed of sound from the thermo-viscoelastic fit^23^ and Eq. (2) from ab initio simulations of liquid Hg with the higher plane-wave cut-off energy. The extrapolation towards $k=0$ results in quite large error bars: the estimated adiabatic speed of sound from the thermo-viscoelastic fit (application of Eq. 2 for $c_s$) is within the range 1140–1200 m/s, that makes evidence for the advantage of the proposed here new methodology for estimation of the macroscopic adiabatic speed of sound in liquids. Finally, for another binary liquid, molten NaCl, at 1262 K simulated by AIMD with 300 particles we were able to obtain good agreement with the experimental value of the adiabatic speed of sound^41^.

The performed calculations of the adiabatic speed of sound in simple and complex liquids showed a robustness of the proposed methodology and good agreement with experimental values. The most advantages of the proposed methodology is in its simplicity—basically one needs only information about the macroscopic stress tensor fluctuations and *k*-dependent fluctuations of the first time derivative of total mass-current (11). Only static correlation functions of these fluctuations are used for estimation of the adiabatic speed of sound. Additionally the great advantage of this methodology is that one can perform all these calculations in a single simulation run. For classical simulations this method should have the same advantages as the Lustig’s formalism^17^, while for ab initio simulations the proposed method can be superior for estimation of the macroscopic adiabatic speed of sound in liquids.

## Methods

Equilibrium classical and ab initio molecular dynamics simulations of liquids were used for generating trajectories of particles $\textbf{r}_j(t)$, their velocities $\textbf{v}_j(t)$ and forces, acting on them $\textbf{F}_j(t)$ along the trajectories. These quantities enter Eq. (11) for spatial-Fourier components of the first time derivative of mass current, and wave vectors $\textbf{k}$ in this expression were sampled using the periodicity of cubic MD-box as $\textbf{k}=2\pi /L(n,l,m)$ with the smallest possible wave number $k_{min}=2\pi /L$, *L* being the MD-box side length and $n,l,m=0,\pm 1,\pm 2, \ldots$. The *k*-dependent correlators (9) were calculated for all possible directions of *k*-vectors with the same absolute value and averaged over them.

Fluctuations of the macroscopic stress tensor components

$$\begin{aligned} \sigma _{\alpha \beta }=\frac{1}{V}\frac{\partial E_{tot}}{\partial \varepsilon _{\alpha \beta }}~, \end{aligned}$$

where $E_{tot}$ is the total energy and $\varepsilon _{\alpha \beta }$—components of strain tensor, are easily estimated from classical simulations with pair interatomic potentials^1^. In the case of ab initio simulations $E_{tot}$ is the total energy within the electron density functional theory, and the much sophisticated calculations of macroscopic stress tensor components were performed as implemented in the VASP (Vienna Ab-initio Simulation Package)^43–46^. The static averages for stress tensor autocorrelations $\psi ^L(0)$ from classical simulations were averaged over 100,000 configurations, while in much time-demanding ab initio simulations the number of averages were not less than 20,000.

Classical MD simulations for supercritical Ar and soft-sphere fluids were performed using a system of 2000 particles in microcanonical ensemble. The parameters of Lennard-Jones potential for supercritical Ar were: $\sigma =3.405$ Å  and $\varepsilon _{LJ}=119.8~K$. For supercritical Ar we studied 9 densities at T = 280 K, while for soft sphere fluids 7 densities were simulated at the temperature $T^* = T/\varepsilon _{SS} = 0.5843$. Parameters for soft-sphere fluids were the same as in^27^. For both types of fluids the interatomic potentials were cut off at $R_{cut}=12$ Å  and corresponding shift was applied to avoid the discontinuity of potential at $R_{cut}$. The values of all densities for simulated supercritical Ar and soft-core fluid are listed in Supplemental material. For soft-core fluids we used the mass of particles equal to the Argon mass. In both cases the time step of 2 fs was used. Upon a proper equilibration for each density a production run over 360,000 time steps was performed for Ar fluids and over 100,000 time steps for soft-core fluid. The conservation of energy was perfect in all the simulations for Lennard-Jones and soft-core fluids: the drift of energy over the whole production run was not larger than 0.005%. All the *k*-dependent correlators were averaged over all possible directions of wave vectors corresponding to the same absolute value. A binary liquid equimolar Kr–Ar mixture was studied by 4000 particles at T = 116 K using the microcanonical ensemble. For Ar–Ar interaction in the binary liquid the same Lennard-Jones potential as for pure Ar fluid were used. For Kr–Kr interaction the Lennard-Jones potentials were $\sigma _{Kr-Kr}=3.633$ Å  and $\varepsilon _{Kr-Kr}=167.0~K$, and parameters for Kr–Ar interaction were $\sigma _{Kr-Ar}=(\sigma _{Kr-Kr}+\sigma _{Ar-Ar})/2$ and $\varepsilon _{Kr-Ar}=\sqrt{\varepsilon _{Kr-Kr}\varepsilon _{Ar-Ar}}$. Similarly, as for pure fluids, the potentials were cut off at $R_{cut}=12$ Å  and correspondingly shifted. The production runs were performed for 360,000 time steps, each of 2 fs.

Ab initio simulations were performed for liquid Sb at T = 973 K with 600 particles and 1800 electron states, for liquid Hg at T = 1750 K with 200 particles and 1300 electron states, and for molten NaCl at T = 1262 K with 300 particles and 750 electron states. In all ab initio simulations the electron-ion interaction were represented by projector-augmented-wave (PAW) potentials, which allow correct representation of the electron density in the core region, and exchange-correlation effects were treated by generalized gradient approximation (GGA) of the energy functional in Perdew-Burke-Ernzerhof^34^ formulation.

### Supplementary Information

Supplementary Information.

## Data Availability

All data generated or analysed during this study are included in this published article and its supplementary information files.
