# Supplementary material for: Stress fluctuations and adiabatic speed of sound in liquids: a simple way to estimate it from ab initio simulations
Source: Sci Rep. 2023 Oct 23;13:18042. doi: 10.1038/s41598-023-45338-2 (PMC10593855; doi:10.1038/s41598-023-45338-2)
Supplement: Supplementary file 1 — Supplementary Information. [file 41598_2023_45338_MOESM1_ESM.pdf]

Supplement material to: "Stress fluctuations and adiabatic speed of sound in liquids: A simple way to estimate it from *ab initio* simulations"

1 Data for estimation of the adiabatic speed of sound in supercritical Ar and Kr-Ar liquid mixture

Fluid and temperature	Density (kg/m^3)	c_∞ (m/s)	$\psi^L(0)$ (GPa)	c_s (m/s)
Ar, 280K	1809.6	2687.0	6.4257	1915.4
	1621.2	2319.5	4.4845	1616.7
	1318.8	1813.8	2.5170	1175.3
	1198.3	1621.7	2.0184	972.4
	1118.0	1519.9	1.6722	902.4
	973.0	1373.9	1.2085	803.5
	600.0	974.7	0.4349	474.6
	395.0	806.2	0.1940	398.5
	186.0	616.4	0.2464	323.2
Kr-Ar, 116K	1832.0	1208.3	1.7553	722.0

Table 1. Mass density, high-frequency speed of sound c_∞, instantaneous value of the longitudinal stress autocorrelation $\psi^L(0)$ and resulting adiabatic speed of sound c_s for supercritical Ar at T=280 K and Kr-Ar mixture at T=116 K.

2 Data for estimation of the adiabatic speed of sound in soft-sphere fluids

Reduced density n^*	c_∞ (m/s)	$\psi^L(0)$ (GPa)	c_s (m/s)	c_s[1] (m/s)
0.9648	2701.85	4.37075	2145.69	2081.41
0.7848	2104.75	2.33524	1630.72	1589.98
0.6351	1658.31	1.27139	1248.52	1225.71
0.4465	1202.08	0.51455	871.28	863.68
0.2976	902.77	0.19676	649.30	638.70
0.1726	685.57	0.06994	478.37	473.06
0.0496	496.99	0.01020	353.04	364.51

Table 2. Reduced density n^*, high-frequency speed of sound c_∞, instantaneous value of the longitudinal stress autocorrelation $\psi^L(0)$ and resulting adiabatic speed of sound c_s for soft sphere fluids at reduced temperature T^*=0.5843. For comparison the values of adiabatic speed of sound c_s, obtained via sampling of heat fluctuations[1] are shown.

3 Data for estimation of the adiabatic speed of sound from *ab initio* simulations

Liquid and temperature	Density (kg/m^3)	c_∞ (m/s)	$\psi^L(0)$ (GPa)	c_s (m/s)
Sb, 973 K	6429.69	3293.93	45.718	1923.9
Hg, 1750 K	10000.00	1784.7	17.629	1192.5
NaCl, 1262 K	1494.7002	4223.74	22.865	1594.56

Table 3. Mass density, high-frequency speed of sound c_∞, instantaneous value of the longitudinal stress autocorrelation $\psi^L(0)$ and resulting adiabatic speed of sound c_s for liquid Sb, Hg and NaCl as obtained from *ab initio* simulations.

References

1. Bryk, T., Gorelli, F., Ruocco, G., Santoro, M. & Scopigno, T. Collective excitations in soft-sphere fluids. *Phys. Rev. E* **90**, 042301, DOI: 10.1103/PhysRevE.90.042301 (2014).
